# Exercise training improves hepatic enzymes, body mass index, and cardiorespiratory fitness in adults with metabolic dysfunction-associated steatotic liver disease: a systematic review and meta-analysis

**DOI:** 10.3389/fphys.2026.1976073

**Published:** 2026-10-16

**Authors:** Sai Long, Yao Li

**Affiliations:** School of Physical Education, Jiangxi Normal University Science and Technology College, Jiujiang, Jiangxi, China

**Keywords:** cardiorespiratory fitness, exercise training, hepatic enzymes, insulin resistance, intrahepatic fat, metabolic dysfunction-associated steatotic liver disease, non-alcoholic fatty liver disease

## Abstract

**Background:**

Exercise is recommended for metabolic dysfunction-associated steatotic liver disease (MASLD). Recent reviews have addressed specific exercise modalities or selected outcome domains, but an updated integrated appraisal of hepatic, metabolic, and cardiorespiratory responses across randomized trials remains needed.

**Method:**

We conducted a PRISMA 2020-compliant systematic review and meta-analysis of randomized controlled trials in adults with MASLD or legacy diagnoses of non-alcoholic fatty liver disease/non-alcoholic steatohepatitis. PubMed, Embase, CENTRAL, Scopus, Web of Science, and SPORTDiscus were searched from inception to June 2026. Post-intervention mean differences (MDs) or standardized mean differences (SMDs) with 95% confidence intervals (CIs) were pooled. Risk of bias was assessed using RoB 2 and certainty using GRADE.

**Results:**

Twenty-five trials were included. Exercise reduced alanine aminotransferase (ALT; MD −7.33 U/L, 95% CI −10.71 to −3.95), aspartate aminotransferase (AST; MD −5.52 U/L, 95% CI −10.24 to −0.79), and BMI (random-effects MD −1.54 kg/m², 95% CI −2.28 to −0.80), and improved VO_2_peak/VO_2_max (MD 4.08 mL/kg/min, 95% CI 1.00 to 7.16). Pooled effects for liver fat/steatosis, HOMA-IR, and HbA1c were not statistically significant. Egger testing showed no significant asymmetry for ALT or AST, whereas BMI showed funnel-plot asymmetry consistent with possible small-study effects.

**Conclusion:**

Exercise training improves hepatic enzymes, BMI, and cardiorespiratory fitness in adults with MASLD/NAFLD, whereas evidence for liver fat and glycemic outcomes remains less certain. PROSPERO: CRD420261416397.

**Systematic review registration:**

https://www.crd.york.ac.uk/PROSPERO/, identifier CRD420261416397.

## 1 Introduction

Metabolic dysfunction-associated steatotic liver disease (MASLD), the current nomenclature for most patients previously classified as having non-alcoholic fatty liver disease (NAFLD), is closely associated with obesity, insulin resistance, type 2 diabetes, and cardiovascular risk (Younossi et al., 2016; Eslam et al., 2020; Rinella et al., 2023; Chalasani et al., 2018; European Association for the Study of the Liver et al., 2016; Cusi et al., 2022). Because MASLD reflects both hepatic and systemic metabolic dysfunction, structured exercise is relevant not only to weight management but also to adaptations in skeletal muscle, substrate utilization, insulin action, cardiorespiratory fitness, and liver-related physiology (Mambrini et al., 2024; Ashcroft et al., 2024).

Randomized trials have evaluated aerobic, resistance, interval, combined, Pilates, yoga, and walking-based programs in adults described as having NAFLD, non-alcoholic steatohepatitis (NASH), MASLD, or metabolic dysfunction-associated steatohepatitis (MASH) (Abdelbasset et al., 2020; Cheng et al., 2017; Hallsworth et al., 2015; Shojaee-Moradie et al., 2016; Sullivan et al., 2012; Zelber-Sagi et al., 2014; Stine et al., 2022; Keating et al., 2023; Zhang et al., 2016; Ezpeleta et al., 2023; Kim et al., 2025). Across this literature, exercise prescriptions, supervision, comparator conditions, diagnostic criteria, and outcome measurement vary substantially, which complicates interpretation of the physiological response to training.

Several recent syntheses have evaluated exercise in NAFLD/MASLD, but they have addressed partly different questions. Huang et al. (2024) used network meta-analysis to compare and rank exercise modalities across metabolic and liver-function outcomes, whereas Arita et al. (2025) combined Mediterranean-diet and exercise interventions with emphasis on anthropometric and liver outcomes. Chen et al. (2025) focused on body composition, blood pressure, and cardiorespiratory fitness in overweight or obese adults with MASLD, and Wang et al. (2026) focused specifically on high-intensity interval training and moderate-intensity continuous training, including feasibility and dose-response analyses. These reviews are informative, but differences in intervention scope, outcome selection, and analytical frameworks leave uncertainty about the pattern of response across liver-specific, metabolic, and cardiorespiratory domains when structured exercise is treated as the common exposure. In addition, several outcomes remain represented by relatively small randomized evidence bases and heterogeneous measurement approaches. An updated pairwise synthesis using a consistent analytical framework is therefore useful for identifying which adaptations are supported most consistently and where uncertainty remains.

This systematic review and meta-analysis therefore quantified the effects of structured exercise training on seven prespecified outcomes in adults with MASLD/NAFLD. ALT and AST were selected as biochemical markers of hepatocellular injury; liver fat/steatosis measures represent the hepatic phenotype more directly; HOMA-IR and HbA1c reflect insulin resistance and longer-term glycemic regulation; BMI reflects overall adiposity burden; and VO_2_peak/VO_2_max reflects cardiorespiratory fitness and exercise adaptation. The latter metabolic and functional outcomes are clinically relevant to the cardiometabolic context of MASLD but should not be interpreted as direct evidence that MASLD itself has improved. By integrating these complementary domains within the same review, the analysis was designed to distinguish liver-specific effects from broader metabolic and cardiorespiratory responses.

## 2 Materials and methods

### 2.1 Study design, protocol, and reporting

This systematic review and meta-analysis was prospectively registered in PROSPERO (CRD420261416397) and conducted in accordance with the PRISMA 2020 statement (Page et al., 2021). The review question was specified using the PICOS framework: adults with MASLD/NAFLD (population), structured exercise training (intervention), non-exercise or matched-control conditions (comparator), hepatic, metabolic, and cardiorespiratory outcomes (outcomes), and randomized controlled trials (study design). The review question, eligibility criteria, and prespecified outcomes were unchanged after registration. During manuscript revision, BMI was re-pooled using a random-effects model and an additional AST sensitivity analysis excluding Kim et al. (2025) was added in response to peer review; these analytical refinements did not alter the eligibility criteria or outcome definitions.

### 2.2 Eligibility criteria

Eligible studies were randomized controlled trials enrolling adults diagnosed with MASLD, NAFLD, NASH, or MASH using accepted clinical, imaging, histological, or contemporaneous diagnostic criteria. Eligible interventions were structured exercise programs with a defined mode, frequency, intensity, duration, or intervention period. Comparators included no exercise, usual care, health education, waiting-list control, or a matched non-exercise co-intervention. Trials were required to report at least one prespecified outcome: alanine aminotransferase (ALT), aspartate aminotransferase (AST), liver fat/steatosis, BMI, homeostatic model assessment for insulin resistance (HOMA-IR), HbA1c, or VO_2_peak/VO_2_max. For the liver-fat synthesis, eligible measurements included direct quantitative imaging approaches such as MRI-derived proton density fat fraction or proton magnetic resonance spectroscopy, as well as controlled attenuation parameter (CAP) obtained by transient elastography. CAP is an indirect marker of hepatic steatosis rather than a direct quantitative measurement of intrahepatic lipid; accordingly, these heterogeneous methods were pooled on the standardized mean difference scale and this measurement heterogeneity was considered when interpreting the result. English-language full-text reports were eligible when group means, standard deviations, and sample sizes were available or could be derived from reported statistics or figures. Studies in children or adolescents, animal or cellular studies, non-randomized or single-arm designs, reviews, letters, case reports, conference abstracts, and interventions in which the independent effect of exercise could not be isolated were excluded. Trials combining exercise with diet were eligible only when the control group received the same dietary component.

### 2.3 Information sources and search strategy

PubMed, Embase, Cochrane CENTRAL, Scopus, Web of Science, and SPORTDiscus were searched from database inception to June 2026. Search concepts covered MASLD/NAFLD/NASH/MASH, exercise and physical training, aerobic and resistance training, high-intensity interval training, and randomized controlled trials. Complete database-specific strategies are provided in **Supplementary File 3**.

### 2.4 Study selection and data extraction

Two reviewers independently screened titles and abstracts and then assessed potentially eligible full texts. Disagreements were resolved by discussion. Extracted variables included author, year, country, participant and diagnostic characteristics, sample size, exercise prescription, comparator, intervention duration, and outcome data. When multiple eligible exercise arms shared one control group, exercise arms were combined for the principal exercise-versus-control synthesis to avoid double-counting the control group. The post-intervention outcome dataset used in the meta-analyses is provided in **Supplementary File 2**.

### 2.5 Risk-of-bias assessment

Risk of bias was assessed using the Cochrane RoB 2 tool for randomized trials (Sterne et al., 2019). Judgments addressed the randomization process, deviations from intended interventions, missing outcome data, measurement of outcomes, and selection of the reported result. The study-level traffic-light plot is provided in **Supplementary Figure S1**.

### 2.6 Certainty of evidence

The certainty of evidence for each meta-analyzed outcome was rated using the GRADE framework (Guyatt et al., 2008), considering risk of bias, inconsistency, imprecision, indirectness, and publication bias. A concise summary is presented in **Table 1** and the domain-level assessment in **Supplementary Table S2**.

**Table 1:** Summary of GRADE certainty for meta-analyzed outcomes.

Outcome	Evidence	Pooled effect (95% CI)	Main reason for downgrading	Certainty
ALT	15 RCTs; n=504	MD −7.33 U/L (95% CI −10.71 to −3.95)	Inconsistency	Moderate
AST	13 RCTs; n=439	MD −5.52 U/L (95% CI −10.24 to −0.79)	Risk of bias and very serious inconsistency	Very low
Liver fat/steatosis	7 RCTs; n=233	SMD −0.36 (95% CI −0.93 to 0.20)	Risk of bias, inconsistency, and imprecision	Very low
BMI	17 RCTs; n=564	MD −1.54 kg/m² (95% CI −2.28 to −0.80)	Possible small-study effects/funnel-plot asymmetry	Moderate
HOMA-IR	6 RCTs; n=218	MD −0.49 (95% CI −1.70 to 0.72)	Risk of bias, inconsistency, and imprecision	Very low
HbA1c	6 RCTs; n=222	MD −0.18% (95% CI −0.52 to 0.16)	Risk of bias, inconsistency, and imprecision	Very low
VO_2_peak/VO_2_max	7 RCTs; n=232	MD 4.08 mL/kg/min (95% CI 1.00 to 7.16)	Inconsistency and imprecision	Low

RCTs begin as high-certainty evidence in GRADE. Detailed domain-level judgments are provided in **Supplementary Table S2**. CI, confidence interval; MD, mean difference; SMD, standardized mean difference.

### 2.7 Data synthesis and statistical analysis

Post-intervention means and standard deviations were used for the main analyses. For continuous outcomes in randomized trials, post-intervention values and change-from-baseline values can both estimate the intervention effect; we used post-intervention values consistently because these data were available across the included trials and this approach avoided imputing the often-unreported pre–post correlation required to derive change-score standard deviations (Higgins et al., 2024). Mean differences (MDs) were calculated for ALT, AST, BMI, HOMA-IR, HbA1c, and VO_2_peak/VO_2_max, whereas standardized mean differences (SMDs) were used for liver fat/steatosis because measurement methods and units differed across trials. Analyses were performed in R (R Foundation for Statistical Computing, Vienna, Austria) using established meta-analytic procedures (Viechtbauer, 2010). Statistical heterogeneity was evaluated using I² and the chi-square test. A common-effect model was used when heterogeneity was low and clinical/methodological heterogeneity was judged negligible; otherwise, a random-effects model was used. Random-effects models were used for ALT, AST, BMI, HOMA-IR, HbA1c, and VO_2_peak/VO_2_max. For BMI, a random-effects model was used in the revised analysis despite low statistical heterogeneity because populations, exercise modalities, comparators, and intervention durations varied across trials. Prediction intervals were reported for random-effects analyses where estimable. Leave-one-out sensitivity analyses were performed for major outcomes, and an additional sensitivity analysis excluded Kim et al. (2025) from the AST synthesis because its effect estimate was substantially larger than those of the other trials. Duration subgroup analyses (≤12 weeks vs >12 weeks) were conducted for ALT and AST. For outcomes with at least 10 studies, funnel plots and Egger regression were used to examine funnel-plot asymmetry/possible small-study effects (Egger et al., 1997). Formal small-study-effect assessment was not performed for outcomes with fewer than 10 studies. Trim-and-fill was used only as an exploratory analysis when Egger testing suggested asymmetry (Duval and Tweedie, 2000). Meta-regression was not performed because only three outcomes included at least 10 studies, limiting reliable moderator analyses across the prespecified outcome set.

## 3 Results

### 3.1 Study selection

The database searches identified 4,694 records: PubMed (n=522), Embase (n=1,188), CENTRAL (n=892), Scopus (n=1,181), Web of Science (n=842), and SPORTDiscus (n=69). After 2,304 duplicates were removed, 2,390 records were screened and 2,304 were excluded. Eighty-six reports were sought for retrieval, 10 could not be retrieved, and 76 full-text reports were assessed. Fifty-one reports were excluded, leaving 25 randomized controlled trials in the systematic review and meta-analysis (**Figure 1**).

**Figure 1:**
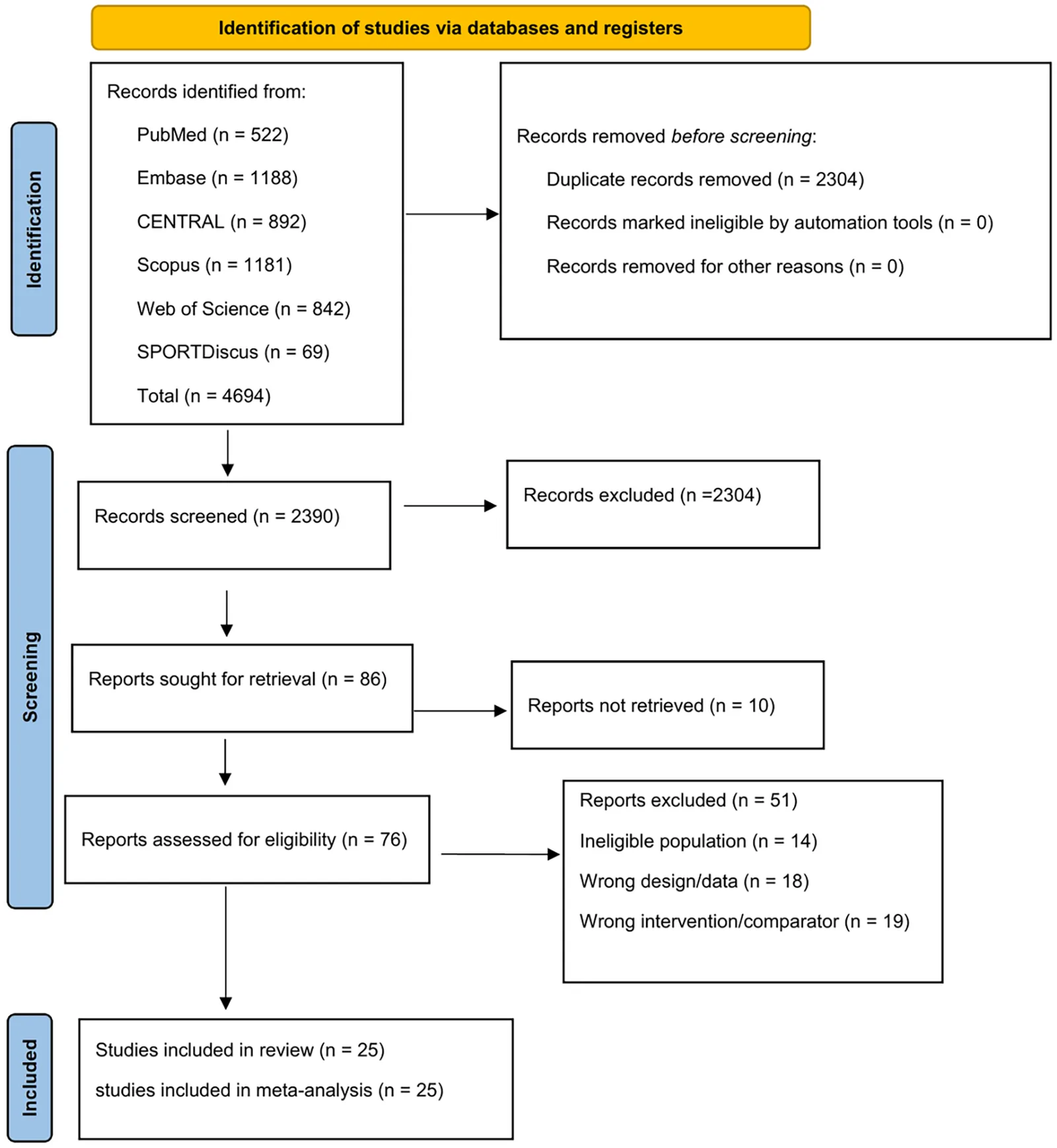
PRISMA 2020 flow diagram for study identification and selection.

### 3.2 Study characteristics

The 25 included trials were published from 2003 to 2026 and represented Asia, Europe, North America, Australia, and the Middle East. Exercise modes included aerobic, resistance, high-intensity interval, combined, Pilates, yoga, and walking-based training, with intervention durations from 6 weeks to 12 months. Detailed participant characteristics, diagnostic approaches, exercise prescriptions, comparators, intervention durations, and available outcomes are provided in **Supplementary Table S1**.

The included trials were reported by Achten et al. (2003); Sullivan et al. (2012); Pugh et al. (2013); Zelber-Sagi et al. (2014); Hallsworth et al. (2015); Shamsoddini et al. (2015); Shojaee-Moradie et al. (2016); Zhang et al. (2016); Cheng et al. (2017); Ghamarchehreh et al. (2019); Abdelbasset et al. (2020); Keymasi et al. (2020); Astinchap et al. (2021); Rajabi et al. (2021); Babu et al. (2022); Stine et al. (2022); Ezpeleta et al. (2023); Hassabi et al. (2023); Keating et al. (2023); Bagnato et al. (2024); Willis et al. (2024); El-Hadidy et al. (2025); Kim et al. (2025); Shi et al. (2025), and Bayat et al. (2026).

### 3.3 Risk of bias

No trial was judged to be at high overall risk of bias in the updated RoB 2 assessment. Approximately half of the studies were judged at low overall risk and the remainder as having some concerns. Concerns most often reflected incomplete reporting of randomization procedures, missing outcome data, or limited prospective information about planned analyses. Outcome measurement was generally low risk because most outcomes were objective laboratory, imaging, or physiological measures. The domain-level percentage summary is shown in **Figure 2** and individual study judgments in **Supplementary Figure S1**.

**Figure 2:**
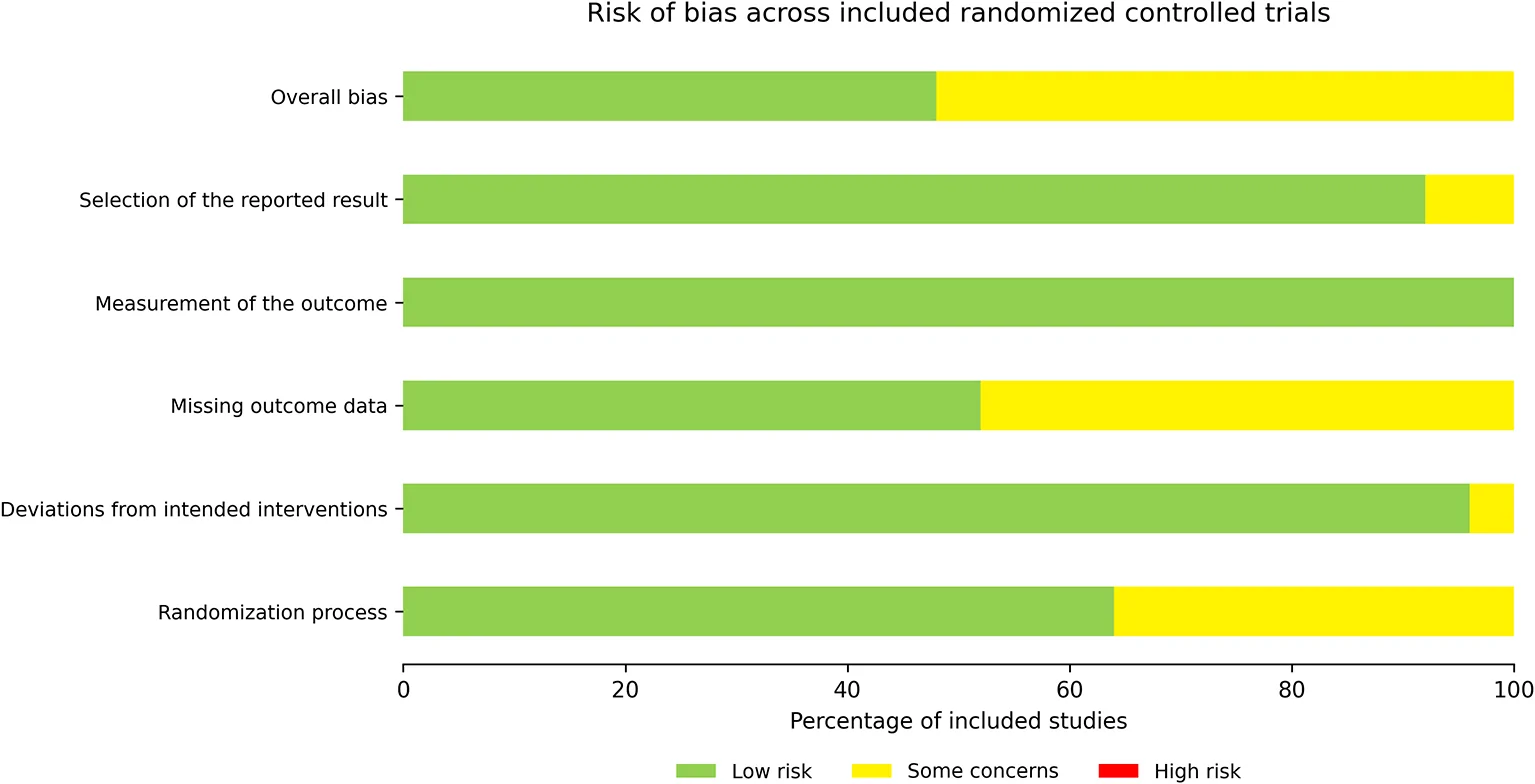
Percentage distribution of RoB 2 judgments across domains and overall risk of bias for the included randomized controlled trials.

### 3.4 Meta-analysis results

#### 3.4.1 Hepatic enzymes

ALT was reported by 15 trials. Using a random-effects model, exercise training reduced post-intervention ALT compared with control (MD −7.33 U/L, 95% CI −10.71 to −3.95; I²=71.3%; **Figure 3**). The prediction interval crossed the null (−18.40 to 3.74), indicating that the magnitude of the effect may vary across future settings. In the duration subgroup analysis, interventions lasting ≤12 weeks showed a larger reduction (MD −10.09 U/L, 95% CI −14.34 to −5.84) than interventions lasting >12 weeks (MD −2.46 U/L, 95% CI −6.13 to 1.21), with a significant subgroup difference (p=0.0011; **Supplementary Figure S5**). AST was reported by 13 trials and, using a random-effects model, was also lower after exercise (MD −5.52 U/L, 95% CI −10.24 to −0.79; I²=93.8%; **Figure 4**). The AST duration subgroup difference was not statistically significant (p=0.0720; **Supplementary Figure S6**). Because Kim et al. (2025) showed an unusually large AST reduction, we conducted an additional sensitivity analysis excluding that trial. The pooled effect remained statistically significant and heterogeneity decreased markedly (12 trials; MD −3.25 U/L, 95% CI −5.16 to −1.35; I²=33.5%; **Supplementary Figure S13**), indicating that the direction of the AST finding was robust although the original magnitude and heterogeneity were strongly influenced by Kim et al. (2025).

**Figure 3:**
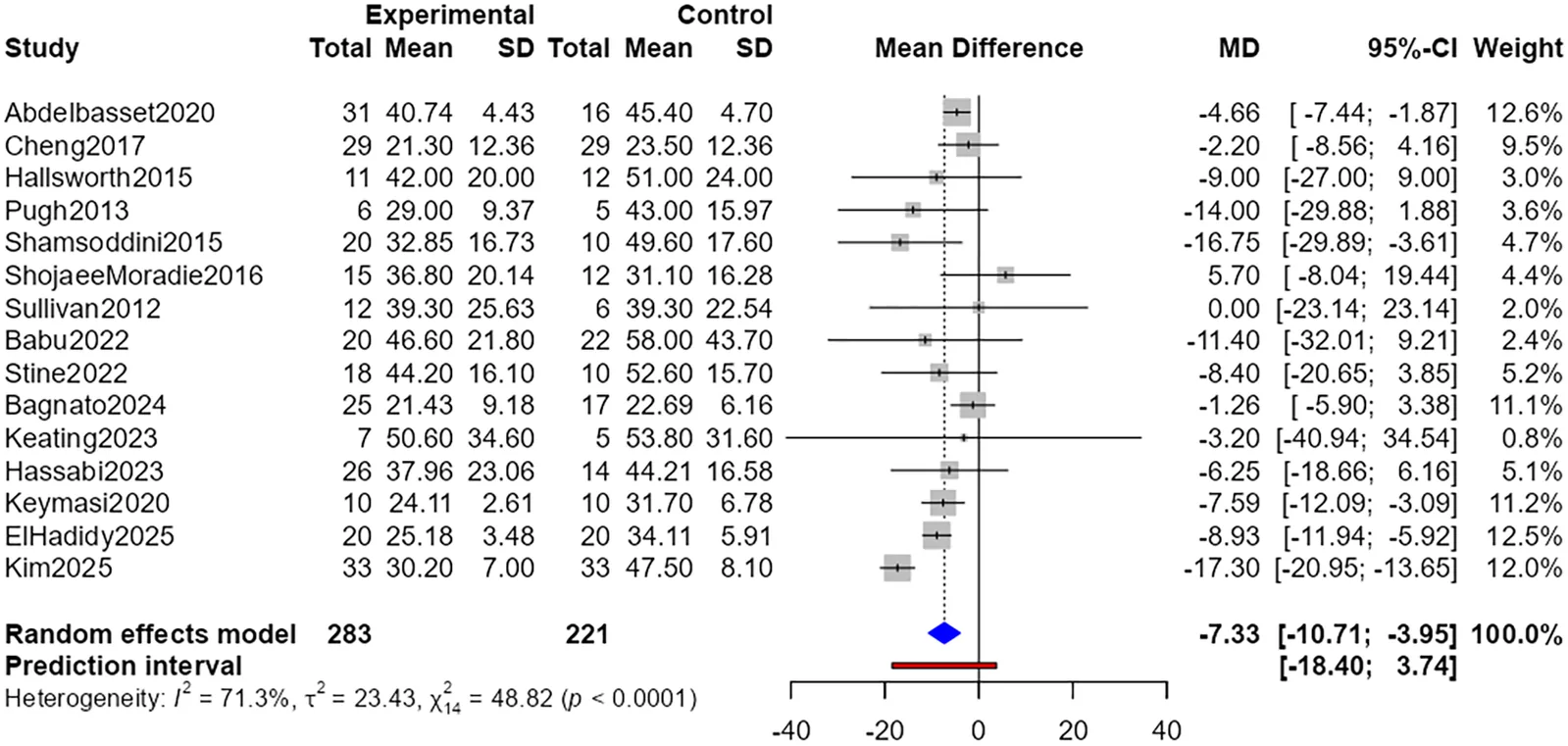
Random-effects forest plot of exercise training effects on post-intervention alanine aminotransferase. Negative values favor exercise training.

**Figure 4:**
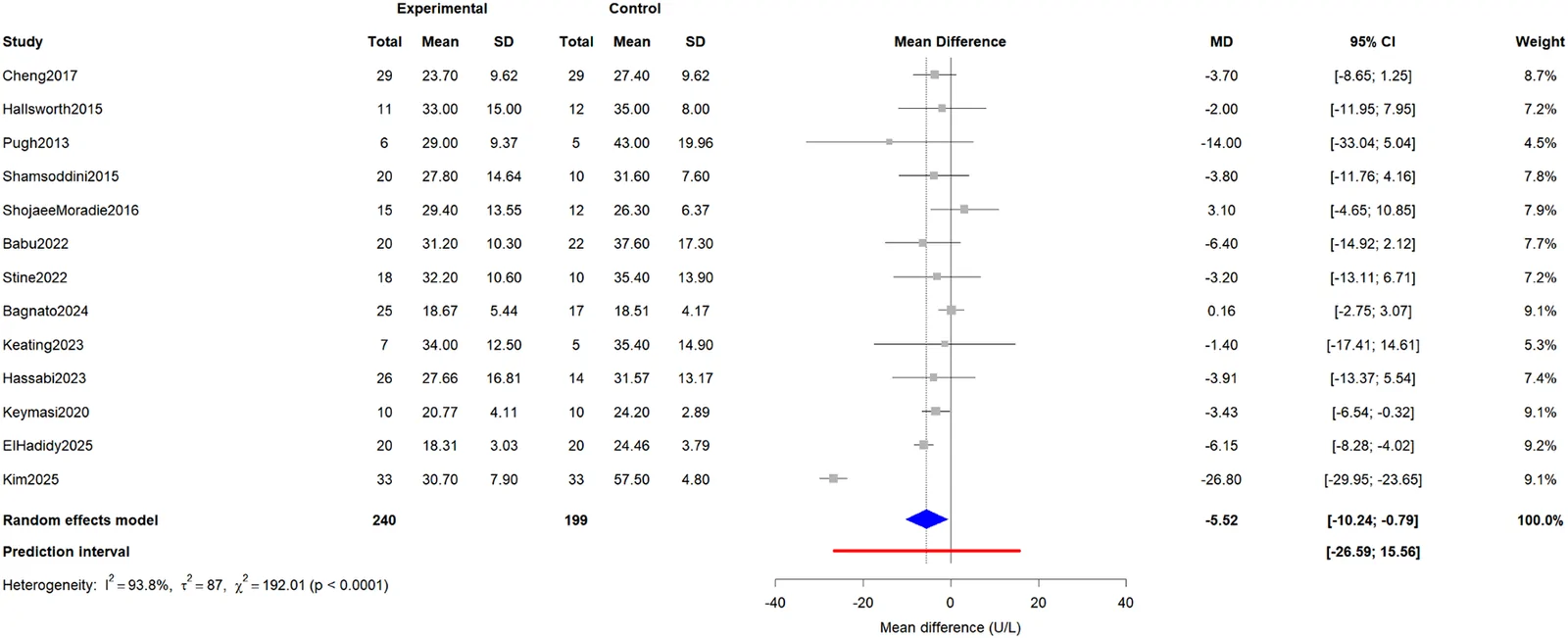
Random-effects forest plot of exercise training effects on post-intervention aspartate aminotransferase. Negative values favor exercise training.

#### 3.4.2 Liver fat/steatosis

Seven trials reported liver fat/steatosis using MRI/MRS-derived direct quantitative measures or CAP obtained by transient elastography. The pooled estimate favored exercise but was not statistically significant (SMD −0.36, 95% CI −0.93 to 0.20; I²=49.9%; **Figure 5**). Because CAP is an indirect steatosis measure and is not directly equivalent to MRI/MRS quantification of intrahepatic lipid, this pooled SMD should be interpreted as a synthesis across related but non-equivalent measurement approaches.

**Figure 5:**
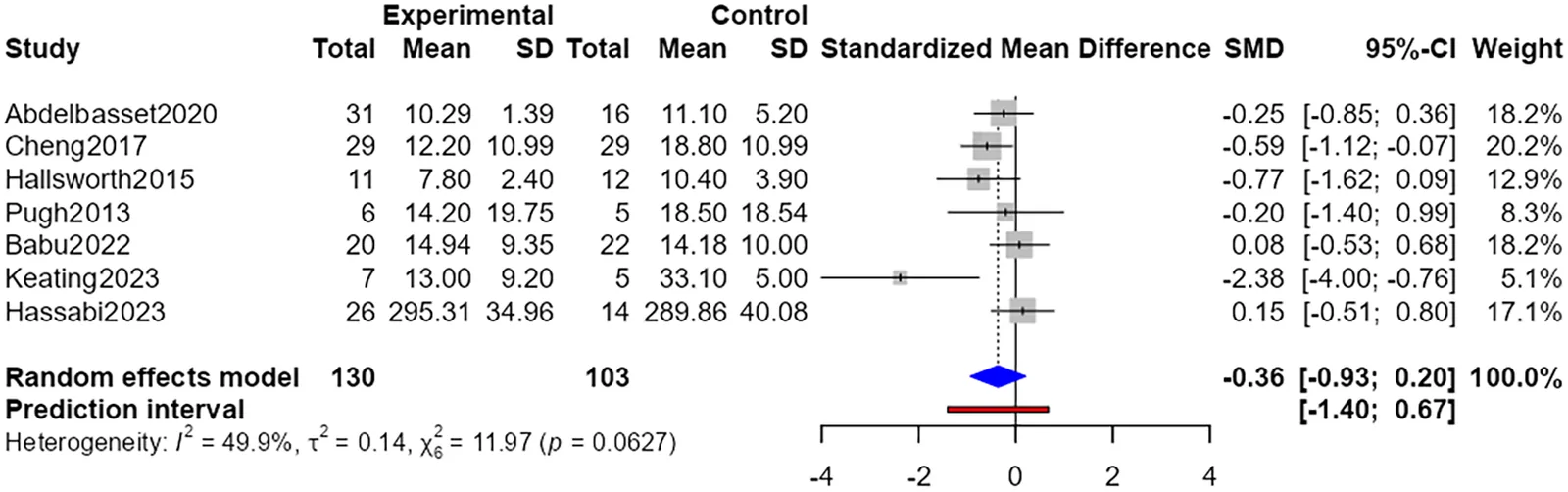
Forest plot of exercise training effects on liver fat/steatosis. Negative values favor exercise training; standardized mean differences were used because measurement methods differed across studies.

#### 3.4.3 Body mass index and glycemic outcomes

BMI was reported by 17 trials. Using a random-effects model to account for clinical and methodological heterogeneity across interventions and populations, exercise reduced BMI (MD −1.54 kg/m², 95% CI −2.28 to −0.80; I²=21.0%; **Figure 6**). The prediction interval crossed the null (−3.17 to 0.08), indicating uncertainty in the effect expected in a new setting. Six trials reported HOMA-IR, for which the pooled estimate from a random-effects model favored exercise but was not statistically significant (MD −0.49, 95% CI −1.70 to 0.72; I²=74.9%; **Figure 7**). HbA1c was reported by six trials and, using a random-effects model, similarly showed a non-significant estimate favoring exercise (MD −0.18%, 95% CI −0.52 to 0.16; I²=65.5%; **Figure 8**).

**Figure 6:**
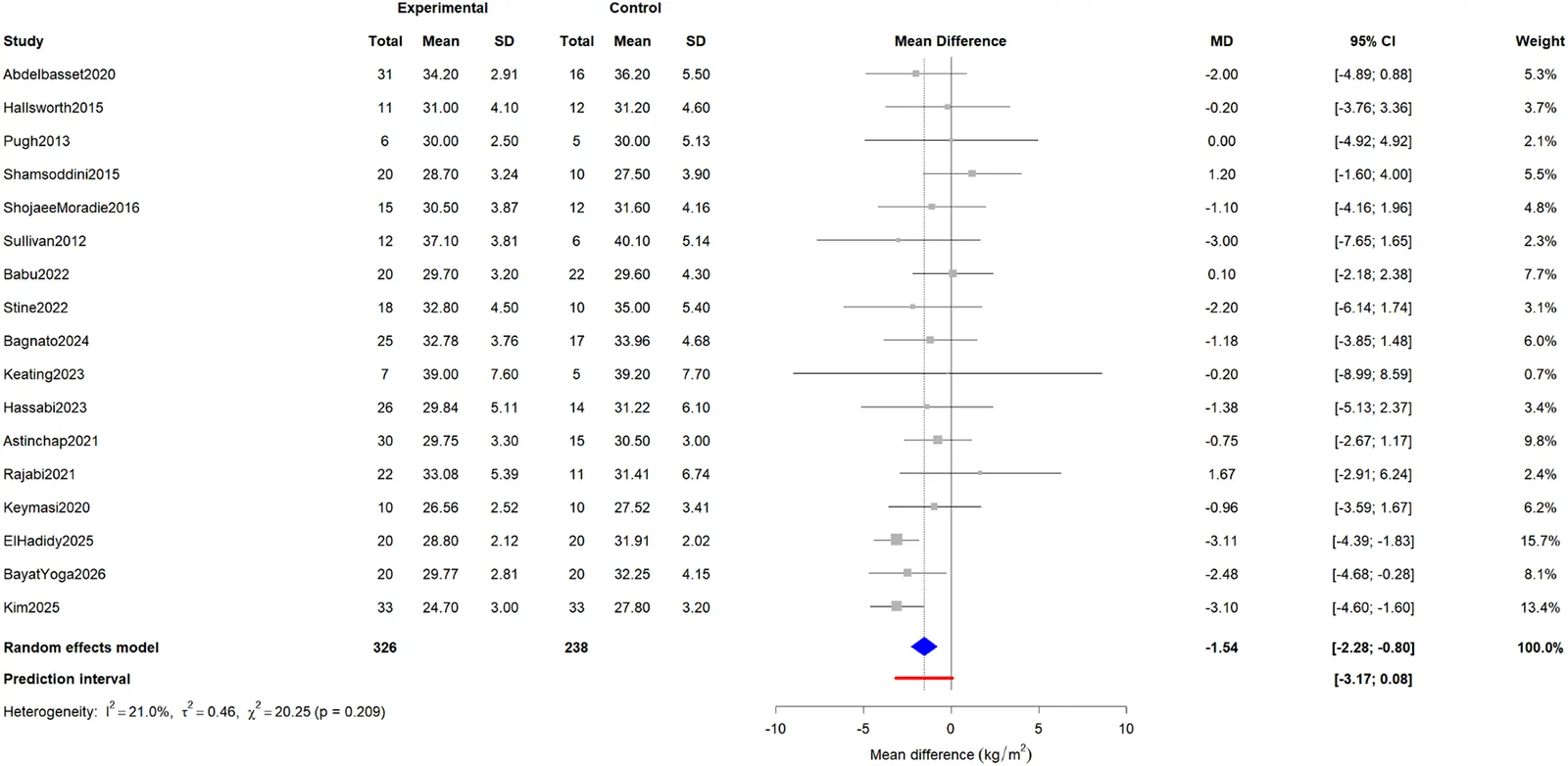
Random-effects forest plot of exercise training effects on post-intervention body mass index. Negative values favor exercise training.

**Figure 7:**
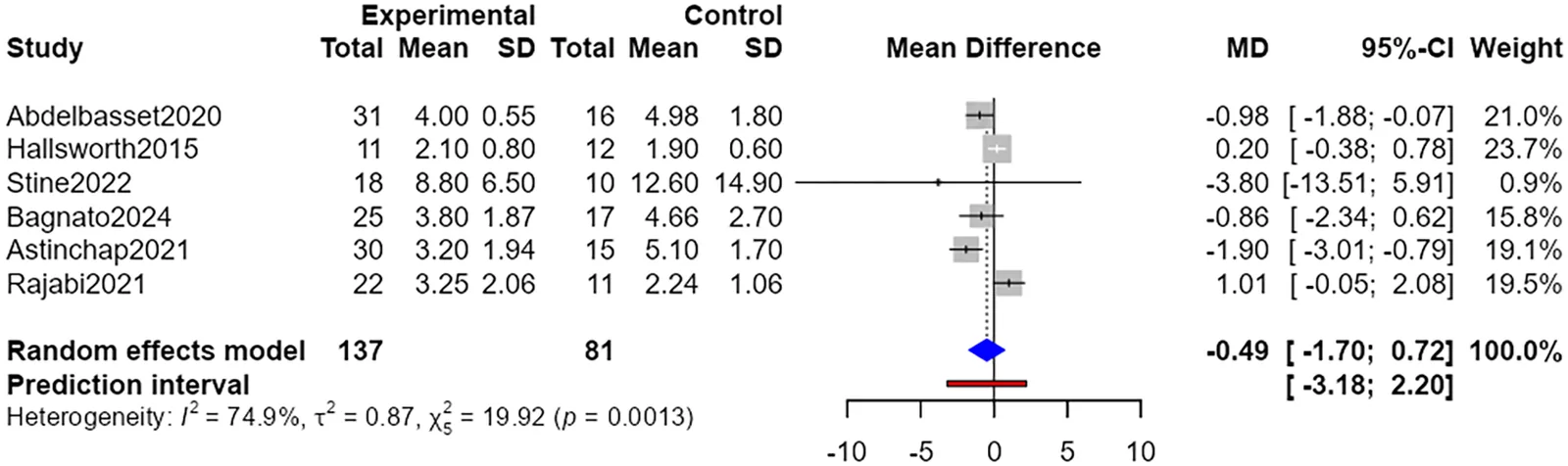
Random-effects forest plot of exercise training effects on post-intervention HOMA-IR. Negative values favor exercise training.

**Figure 8:**
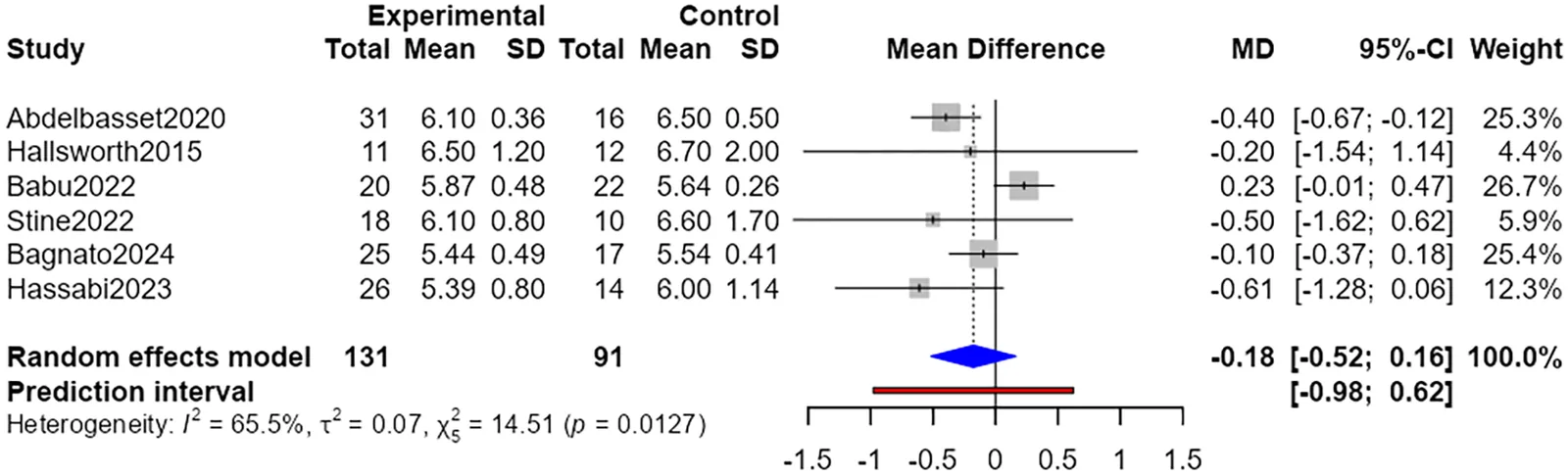
Random-effects forest plot of exercise training effects on post-intervention HbA1c. Negative values favor exercise training.

#### 3.4.4 Cardiorespiratory fitness

Seven trials reported VO_2_peak/VO_2_max. Using a random-effects model, exercise increased cardiorespiratory fitness (MD 4.08 mL/kg/min, 95% CI 1.00 to 7.16; I²=72.3%; **Figure 9**), although the prediction interval crossed the null (−3.91 to 12.08).

**Figure 9:**
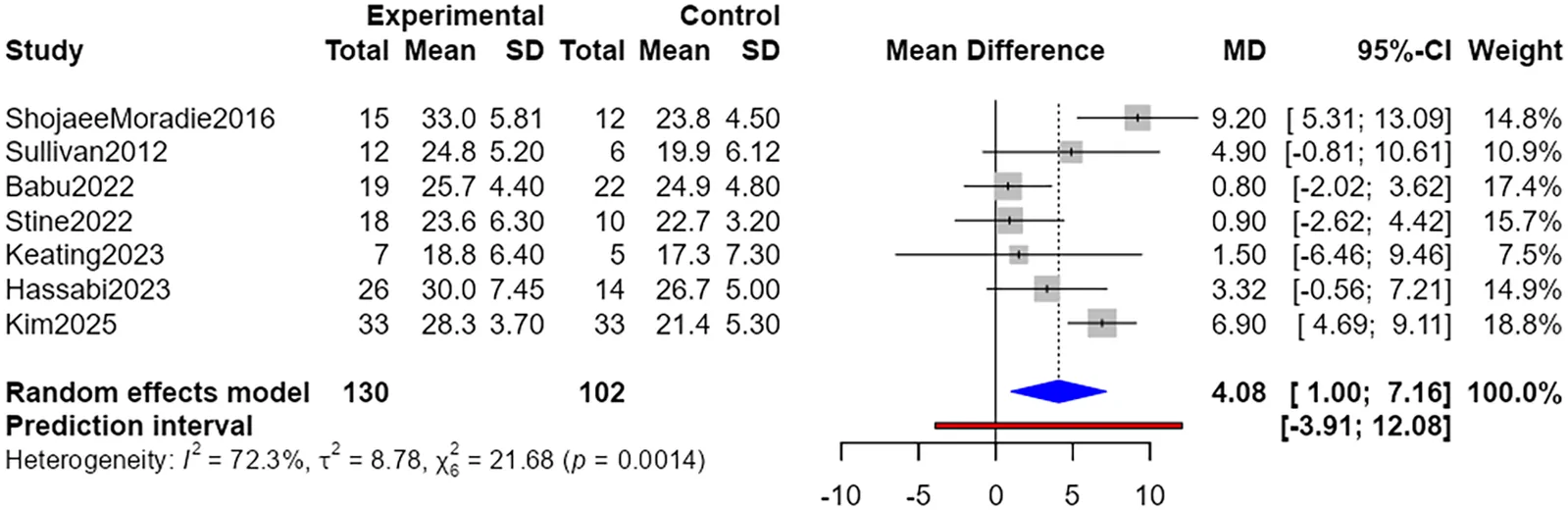
Random-effects forest plot of exercise training effects on post-intervention VO_2_peak/VO_2_max. Positive values favor exercise training.

### 3.5 Sensitivity analyses and small-study effects

Leave-one-out analyses generally preserved the direction of the pooled estimates. ALT and VO_2_peak/VO_2_max remained directionally favorable after omission of individual trials, whereas liver fat/steatosis, HOMA-IR, and HbA1c remained non-significant overall (**Supplementary Figures S7**–**S12**). For AST, the dedicated sensitivity analysis excluding Kim et al. (2025) retained a statistically significant pooled reduction while reducing I² from 93.8% to 33.5% (**Supplementary Figure S13**). Funnel plots and Egger regression were examined only for ALT, AST, and BMI because these outcomes included at least 10 studies (**Supplementary Figures S2**–**S4**). Egger regression did not indicate significant asymmetry for ALT (p=0.812) or AST (p=0.690), whereas BMI showed significant funnel-plot asymmetry (p=0.018), consistent with possible small-study effects. This was not interpreted as definitive publication bias because asymmetry can arise from several sources and the BMI dataset included small, imprecise trials such as Keating et al. (2023). Trim-and-fill was therefore treated as exploratory only. No formal publication-bias or funnel-asymmetry assessment was performed for outcomes with fewer than 10 studies.

### 3.6 Certainty of evidence

GRADE certainty was moderate for ALT and BMI, very low for AST, liver fat/steatosis, HOMA-IR, and HbA1c, and low for VO_2_peak/VO_2_max (**Table 1**). The revised outcome-specific risk-of-bias judgments explicitly considered the proportion and influence of trials rated as having some concerns in RoB 2 rather than assigning ‘Not serious’ uniformly. Downgrading otherwise reflected inconsistency, imprecision, and, for BMI, possible small-study effects/funnel-plot asymmetry. Domain-level judgments and rationales are provided in **Supplementary Table S2**.

## 4 Discussion

### 4.1 Summary of main findings

Across 25 randomized controlled trials, structured exercise training improved several outcomes that reflect integrated hepatic, metabolic, and cardiorespiratory adaptation. Exercise reduced ALT, AST, and BMI and increased VO_2_peak/VO_2_max. Liver fat/steatosis, HOMA-IR, and HbA1c moved in a direction favoring exercise but did not reach statistical significance in the pooled analyses.

### 4.2 Physiological interpretation

The concurrent improvements in hepatic enzymes and cardiorespiratory fitness are consistent with the view that exercise produces multi-system physiological adaptation rather than acting through body-mass change alone. ALT and AST are markers of hepatocellular injury rather than direct measures of hepatic function, so their reductions should be interpreted as biochemical improvement rather than evidence of histological resolution. Related trials have shown that exercise can alter intrahepatic lipid, insulin sensitivity, imaging-derived liver measures, and histological or metabolic surrogates (Bacchi et al., 2013; Houghton et al., 2017; Oh et al., 2017; O'Gorman et al., 2020; Huber et al., 2019; Cuthbertson et al., 2024; Mucinski et al., 2024; Houttu et al., 2025). In the present synthesis, however, the pooled liver-fat/steatosis estimate remained imprecise and combined non-equivalent direct and indirect measurement approaches, indicating that evidence for this adaptation is less consistent than the evidence for enzyme, BMI, and cardiorespiratory outcomes. Improvements in BMI, HOMA-IR, HbA1c, or VO_2_peak/VO_2_max are clinically relevant in the cardiometabolic context of MASLD but are systemic or functional outcomes and should not be interpreted as direct evidence of improvement in MASLD itself. Training-related gains in aerobic capacity are biologically compatible with improved skeletal-muscle oxidative capacity, glucose disposal, fatty-acid utilization, and whole-body metabolic flexibility (Mambrini et al., 2024; Ashcroft et al., 2024). These adaptations may reduce metabolic substrate delivery to the liver, but the non-significant pooled effects for HOMA-IR and HbA1c show that improvements were not uniform across metabolic domains. This separation between biochemical, structural, metabolic, and fitness responses supports evaluating multiple physiological domains rather than treating any single outcome as a surrogate for overall disease resolution.

### 4.3 Comparison with previous evidence

The present findings broadly agree with recent evidence that structured exercise benefits important clinical and physiological outcomes in MASLD, while adding a different analytical perspective. Huang et al. (2024) reported favorable effects of several exercise modalities on BMI, lipid profiles, and liver enzymes in a network meta-analysis, but its primary aim was to rank modalities. Arita et al. (2025) synthesized Mediterranean diet and exercise interventions and found benefits for selected anthropometric and liver outcomes. Chen et al. (2025) reported reductions in BMI and improvements in maximal oxygen uptake in overweight or obese adults with MASLD, which is consistent with the BMI and VO_2_peak/VO_2_max findings in the present analysis. More recently, Wang et al. (2026) reported benefits of both high-intensity interval and moderate-intensity continuous training across anthropometric, metabolic, and liver-related outcomes and explored exercise-dose moderators. Our review complements these studies by treating structured exercise as the common exposure and applying one post-intervention framework across seven prespecified domains: ALT, AST, liver fat/steatosis, BMI, HOMA-IR, HbA1c, and VO_2_peak/VO_2_max. The inclusion of prediction intervals, leave-one-out analyses, small-study-effect testing when sufficiently powered, and GRADE helps distinguish the average pooled effect from the certainty and generalizability of that effect. In particular, the wide prediction intervals for ALT, AST, and VO_2_peak/VO_2_max indicate that an average benefit does not imply a uniform response in every clinical setting, while the non-significant pooled estimates for liver fat and glycemic markers caution against assuming that biochemical or fitness improvements necessarily translate into parallel structural or glycemic changes.

### 4.4 Heterogeneity and subgroup findings

Heterogeneity was substantial for ALT, AST, HOMA-IR, HbA1c, and VO_2_peak/VO_2_max. Differences in baseline disease severity, exercise mode and intensity, supervision, adherence, coexisting metabolic disease, intervention duration, and outcome measurement are plausible contributors. The ALT duration subgroup suggested a larger reduction with interventions lasting ≤12 weeks, but this finding should not be interpreted as evidence that shorter programs are physiologically superior. This between-study subgroup comparison is observational: intervention duration may be associated with baseline ALT, exercise mode and intensity, supervision, adherence, comorbidity, and other differences in trial composition. For example, trials enrolling participants with higher baseline ALT may have greater scope for an absolute reduction, while differences in training delivery and adherence may influence the exercise exposure achieved. These are possible explanations rather than moderators established by the present analysis; duration may therefore act as a proxy for such differences. In addition, the estimate for interventions lasting >12 weeks was imprecise and its 95% CI crossed the null. The significant ALT subgroup difference should consequently be considered hypothesis-generating rather than evidence of a true duration–response relationship. For AST, Kim et al. (2025) was a major source of heterogeneity: excluding this trial reduced I² from 93.8% to 33.5% while the pooled reduction remained statistically significant. This sensitivity result supports the direction of the AST association but also shows that its magnitude is sensitive to an influential trial. The absence of a significant AST duration interaction in the primary subgroup analysis further reinforces cautious interpretation.

### 4.5 Strengths and limitations

This review focused on randomized controlled trials, applied a consistent post-intervention data strategy, combined eligible multi-arm exercise groups to avoid double-counting controls, and integrated hepatic, metabolic, and cardiorespiratory outcomes. RoB 2, leave-one-out analyses, small-study-effect assessments, and GRADE were used to support transparent interpretation. Several limitations remain. Exercise mode, intensity, frequency, supervision, and duration varied substantially. The use of post-intervention values improved consistency across studies and avoided imputing unavailable pre–post correlations, but small randomized trials may still have baseline imbalances that are not fully addressed by an unadjusted final-value comparison. Some outcomes were available from fewer than 10 studies, precluding formal assessment of funnel-plot asymmetry/small-study effects. Liver fat/steatosis was measured using non-equivalent methods: MRI/MRS provide direct quantitative estimates of intrahepatic lipid, whereas CAP is an indirect marker of steatosis; pooling these measures with SMDs does not eliminate this measurement heterogeneity. The review was restricted to English-language full-text reports, so language bias cannot be excluded. In addition, only three outcomes included at least 10 studies; therefore, meta-regression was not used and residual heterogeneity related to exercise dose, baseline disease severity, or comorbidity could not be modeled reliably across outcomes. Finally, several trials enrolled participants with type 2 diabetes, obesity, obstructive sleep apnea, or metabolic syndrome, which may have modified the physiological response to training.

### 4.6 Implications for research and practice

The findings support structured exercise as a physiologically relevant component of MASLD management, particularly when the goals include improving cardiorespiratory fitness and reducing elevated liver enzymes and BMI. Future trials should report exercise dose and adherence in sufficient detail, use standardized and sensitive measures of intrahepatic fat, and prespecify metabolic and cardiorespiratory outcomes. Larger trials are also needed to determine whether specific combinations of exercise mode, intensity, and duration produce reproducible hepatic adaptations independent of weight loss.

## 5 Conclusion

Exercise training is associated with improvements in hepatic enzymes, BMI, and cardiorespiratory fitness in adults with MASLD/NAFLD, while pooled effects on liver fat/steatosis, HOMA-IR, and HbA1c remain uncertain. The AST association remained statistically significant after exclusion of the influential Kim et al. (2025) trial, although the sensitivity analysis showed that the magnitude and heterogeneity of the primary AST estimate were strongly affected by that study. BMI and cardiorespiratory improvements should be viewed as clinically relevant systemic adaptations rather than direct evidence of MASLD resolution. Marked heterogeneity across several outcomes indicates that the optimal exercise prescription for hepatic and metabolic adaptation is not yet established.

## Data Availability

The original contributions presented in the study are included in the article/**Supplementary Material**. Further inquiries can be directed to the corresponding author.

## Supplementary material

Supplementary File 1 Contains the study characteristics table, detailed GRADE assessment, risk-of-bias traffic-light plot, funnel plots, duration subgroup analyses, leave-one-out sensitivity analyses, and the dedicated AST sensitivity analysis excluding Kim et al. (2025)

Supplementary File 2 Contains the extracted post-intervention outcome dataset.

Supplementary File 3 Contains the complete database-specific search strategies.
