# Supplementary material for: Exercise training improves hepatic enzymes, body mass index, and cardiorespiratory fitness in adults with metabolic dysfunction-associated steatotic liver disease: a systematic review and meta-analysis
Source: Front Physiol. 2026 Oct 16;17:1976073. doi: 10.3389/fphys.2026.1976073 (PMC13719568; doi:10.3389/fphys.2026.1976073)
Supplement: Supplementary file 1 [file DataSheet1.zip › Supplementary/Supplementary_File_1_revised.docx]

**Supplementary Material**

# Supplementary Table S1. Characteristics of included randomized controlled trials

| **Study** | **Country** | **Participants / diagnosis** | **Sample size** | **Exercise intervention** | **Comparator** | **Duration** | **Relevant outcomes** |
| --- | --- | --- | --- | --- | --- | --- | --- |
| Abdelbasset et al. (2020) | Egypt/Saudi Arabia | Adults with type 2 diabetes, obesity, and NAFLD; imaging/clinical NAFLD | 47 (E31/C16) | High-intensity interval and moderate-intensity continuous aerobic exercise; exercise arms combined | No-exercise control | 8 weeks | ALT, liver fat, BMI, HOMA-IR, HbA1c |
| Cheng et al. (2017) | China | Adults aged 50-65 years with prediabetes and NAFLD; 1H-MRS liver fat >5% | 58 (E29/C29) | Aerobic exercise | No intervention | 8.6 months | ALT, AST, quantitative liver fat |
| Hallsworth et al. (2015) | UK | Adults with NAFLD; clinical diagnosis and MRI-assessed liver fat | 23 (E11/C12) | Modified high-intensity interval training | Standard care | 12 weeks | ALT, AST, liver fat, BMI, HOMA-IR, HbA1c |
| Pugh et al. (2013) | UK | Adults with NAFLD; clinical/imaging diagnosis | 11 (E6/C5) | Supervised exercise training | Conventional care | 16 weeks | ALT, AST, liver fat, BMI |
| Shamsoddini et al. (2015) | Iran | Adult men with NAFLD; ultrasound diagnosis | 30 (E20/C10) | Aerobic and resistance training; exercise arms combined | Control | 8 weeks | ALT, AST, BMI |
| Shojaee-Moradie et al. (2016) | UK | Adult men with NAFLD; MRS-assessed liver fat | 27 (E15/C12) | Supervised aerobic exercise | Conventional lifestyle advice | 16 weeks | ALT, AST, BMI, VO2max |
| Sullivan et al. (2012) | USA | Adults with obesity and NAFLD; MRS-assessed intrahepatic triglyceride | 18 (E12/C6) | Exercise training | Observation control | 16 weeks | ALT, BMI, VO2peak |
| Zelber-Sagi et al. (2014) | Israel | Adults with NAFLD; ultrasound diagnosis | 64 (E33/C31) | Resistance training | Home stretching control | 12 weeks | Liver fat/steatosis, ALT, AST, BMI, HOMA-IR, HbA1c |
| Babu et al. (2022) | Finland | Adults with NAFLD; MRI/clinical diagnosis | 42 (E20/C22) | High-intensity interval training | Sedentary lifestyle control | 12 weeks | ALT, AST, liver fat, BMI, HbA1c, VO2max |
| Rajabi et al. (2021) | Iran | Women with NAFLD; clinical/ultrasound diagnosis | 33 (E22/C11) | Resistance training combined with aerobic interval or high-intensity interval training; exercise arms combined | Control | 8 weeks | BMI, HOMA-IR, body composition |
| Achten et al. (2003) | Netherlands/UK | Male patients with NASH | 9 analyzed | Supervised aerobic exercise | Non-exercise control | 12 weeks | ALT, body weight, exercise capacity |
| Stine et al. (2022) | USA | Adults with biopsy-confirmed NASH | 28 (E18/C10) | Aerobic exercise | Standard clinical care | 20 weeks | ALT, AST, MRI-PDFF, BMI, HOMA-IR, HbA1c, VO2peak |
| Bagnato et al. (2024) | Italy | Adults with MASLD; clinical/imaging diagnosis | 42 (E25/C17) | Aerobic exercise plus dietary advice and HIIT plus dietary advice; exercise arms combined | Dietary advice alone | 12 weeks | ALT, AST, BMI, HOMA-IR, HbA1c |
| Keating et al. (2023) | Australia | Adults with NASH | 12 (E7/C5) | High-intensity interval training | Sham stretching control | 12 weeks | ALT, AST, quantitative liver fat, BMI, VO2peak |
| Zhang et al. (2016) | China | Adults with NAFLD; 1H-MRS intrahepatic triglyceride | 220 (E146/C74) | Vigorous-moderate and moderate exercise; exercise arms combined | No-exercise control | 12 months | ALT, AST, intrahepatic triglyceride, BMI, metabolic outcomes |
| Willis et al. (2024) | UK | Men with MASLD; MRI-PDFF assessment | 24 (E11/C13); liver-fat analysis n=19 | Exercise training | Non-exercise control | 6 weeks | ALT, AST, PDFF, BMI, HbA1c, HOMA-IR, VO2peak |
| Ghamarchehreh et al. (2019) | Iran | Older adults with NAFLD | 28 completed (three-arm trial) | Aerobic and resistance exercise | Control | 8 weeks | BMI and lipid/metabolic outcomes |
| Hassabi et al. (2023) | Iran | Adults with NAFLD; transient elastography assessment | 40 (E26/C14) | Moderate- and high-intensity aerobic exercise; exercise arms combined | Routine lifestyle management control | 6 months | ALT, AST, CAP/liver fat, BMI, HbA1c, VO2max |
| Ezpeleta et al. (2023) | USA | Adults with obesity and NAFLD; MRI-PDFF intrahepatic triglyceride >5% | 40 for eligible exercise-only comparison (E20/C20) | Exercise alone | No-intervention control | 3 months | ALT, AST, intrahepatic triglyceride, BMI, HOMA-IR, HbA1c |
| Astinchap et al. (2021) | Iran | Women with type 2 diabetes and NAFLD | 45 (E30/C15) | Endurance and resistance training; exercise arms combined | Control | 8 weeks | BMI, HOMA-IR, ultrasound fatty liver grade |
| Shi et al. (2025) | China | Young adults with NAFLD; ultrasound/clinical diagnosis | 40 (AT10/RT10/AT+RT10/C10) | Aerobic, resistance, and combined aerobic-resistance exercise | Dietary control/no exercise | 12 weeks | ALT, AST, BMI, glucose/lipid and bile acid outcomes |
| Keymasi et al. (2020) | Iran | Middle-aged men with NAFLD; ultrasound diagnosis | 20 (E10/C10) | Pilates training | Control | 8 weeks | ALT, AST, liver fat content, BMI |
| El-Hadidy et al. (2025) | Egypt | Patients with obstructive sleep apnea and NAFLD; ultrasound diagnosis | 40 (E20/C20) | CPAP plus free walking exercise | CPAP alone | 12 weeks | ALT, AST, BMI |
| Bayat et al. (2026) | Iran | Women with type 2 diabetes and NAFLD; ultrasound diagnosis | 40 (E20/C20) | Cyclic yoga | Usual activities/control | 8 weeks | BMI and metabolic outcomes |
| Kim et al. (2025) | Korea | Older adults with metabolic syndrome and MASLD; ultrasound/MASLD criteria | 66 (E33/C33) | Treadmill walking programme | Control/meditation | 12 weeks | ALT, AST, BMI, VO2max, ultrasound score |

Note: Sample size refers to the eligible comparison used in the review. When multiple eligible exercise arms shared one control group, exercise arms were combined for the overall exercise-versus-control synthesis.

# Supplementary Table S2. Detailed GRADE assessment

| **Outcome** | **Evidence** | **Effect estimate** | **Risk of bias** | **Inconsistency** | **Imprecision** | **Indirectness** | **Publication bias** | **Certainty** |
| --- | --- | --- | --- | --- | --- | --- | --- | --- |
| ALT | 15 RCTs (504 participants) | MD -7.33 U/L (95% CI -10.71 to -3.95) | Not serious: no high-risk trials; concerns did not dominate the ALT estimate. | Serious | Not serious | Not serious | No significant Egger test (p=0.812) | Moderate |
| AST | 13 RCTs (439 participants) | MD -5.52 U/L (95% CI -10.24 to -0.79) | Serious: several trials had some concerns; influential Kim 2025 also had some concerns. | Very serious | Not serious | Not serious | No significant Egger test (p=0.690) | Very low |
| Liver fat/steatosis | 7 RCTs (233 participants) | SMD -0.36 (95% CI -0.93 to 0.20) | Serious: 4/7 contributing trials had some concerns. | Serious | Serious | Not serious | Not assessed (<10 studies) | Very low |
| BMI | 17 RCTs (564 participants) | MD -1.54 kg/m² (95% CI -2.28 to -0.80) | Not serious: no high-risk trials; the estimate was not dominated by trials with concerns. | Not serious | Not serious | Not serious | Possible small-study effects / funnel asymmetry (Egger p=0.018); not definitive publication bias. | Moderate |
| HOMA-IR | 6 RCTs (218 participants) | MD -0.49 (95% CI -1.70 to 0.72) | Serious: 3/6 contributing trials had some concerns. | Serious | Serious | Not serious | Not assessed (<10 studies) | Very low |
| HbA1c | 6 RCTs (222 participants) | MD -0.18% (95% CI -0.52 to 0.16) | Serious: 4/6 contributing trials had some concerns. | Serious | Serious | Not serious | Not assessed (<10 studies) | Very low |
| VO₂peak/VO₂max | 7 RCTs (232 participants) | MD 4.08 mL/kg/min (95% CI 1.00 to 7.16) | Not serious: no high-risk trials; most evidence was low risk. | Serious | Serious | Not serious | Not assessed (<10 studies) | Low |

Note: RCTs start as high-certainty evidence in GRADE. Risk-of-bias judgments were made at the outcome level using the contributing studies shown in Supplementary Figure S1; 'some concerns' did not automatically trigger downgrading, but risk of bias was downgraded when concerns affected a substantial proportion of the evidence or an influential study. Funnel-plot asymmetry was formally assessed only for outcomes with at least 10 studies. CI, confidence interval; MD, mean difference; SMD, standardized mean difference.

## Supplementary Figure S1. Risk-of-bias traffic-light plot for individual randomized controlled trials.

## Supplementary Figure S2. Funnel plot for alanine aminotransferase.

## Supplementary Figure S3. Funnel plot for aspartate aminotransferase.

## Supplementary Figure S4. Funnel plot for body mass index.

## Supplementary Figure S5. Subgroup analysis of alanine aminotransferase by intervention duration.

## Supplementary Figure S6. Subgroup analysis of aspartate aminotransferase by intervention duration.

## Supplementary Figure S7. Leave-one-out sensitivity analysis for alanine aminotransferase.

## Supplementary Figure S8. Leave-one-out sensitivity analysis for aspartate aminotransferase.

## Supplementary Figure S9. Leave-one-out sensitivity analysis for liver fat/steatosis.

## Supplementary Figure S10. Leave-one-out sensitivity analysis for HOMA-IR.

## Supplementary Figure S11. Leave-one-out sensitivity analysis for HbA1c.

## Supplementary Figure S12. Leave-one-out sensitivity analysis for VO₂peak/VO₂max.

## Supplementary Figure S13. Sensitivity analysis of post-intervention aspartate aminotransferase excluding Kim et al. (2025). The pooled reduction remained statistically significant (MD −3.25 U/L, 95% CI −5.16 to −1.35) and heterogeneity decreased to I²=33.5%. Negative values favor exercise training.
