# Supplementary material for: Exercise training improves hepatic enzymes, body mass index, and cardiorespiratory fitness in adults with metabolic dysfunction-associated steatotic liver disease: a systematic review and meta-analysis
Source: Front Physiol. 2026 Oct 16;17:1976073. doi: 10.3389/fphys.2026.1976073 (PMC13719568; doi:10.3389/fphys.2026.1976073)
Supplement: Supplementary file 1 [file DataSheet1.zip › Supplementary/Supplementary_File_3_Search_Strategies.pdf]

Supplementary File 3. Complete database-specific search strategies

Searches were conducted from database inception to June 2026.

PubMed (n=522)

```
(("Non-alcoholic Fatty Liver Disease"[Mesh]
OR "non-alcoholic fatty liver disease"[tiab]
OR "nonalcoholic fatty liver disease"[tiab]
OR NAFLD[tiab]
OR "metabolic dysfunction-associated steatotic liver disease"[tiab]
OR "metabolic dysfunction associated steatotic liver disease"[tiab]
OR MASLD[tiab]
OR "non-alcoholic steatohepatitis"[tiab]
OR "nonalcoholic steatohepatitis"[tiab]
OR NASH[tiab]
OR "metabolic dysfunction-associated steatohepatitis"[tiab]
OR MASH[tiab]
OR "hepatic steatosis"[tiab]
OR "liver fat"[tiab])

AND

("Exercise"[Mesh]
OR "Exercise Therapy"[Mesh]
OR exercise[tiab]
OR "exercise training"[tiab]
OR "physical training"[tiab]
OR "aerobic training"[tiab]
OR "resistance training"[tiab]
OR "strength training"[tiab]
OR "endurance training"[tiab]
OR "high-intensity interval training"[tiab]
OR HIIT[tiab]
OR "combined training"[tiab])

AND

("Randomized Controlled Trial"[Publication Type]
OR randomized[tiab]
OR randomised[tiab]
OR randomly[tiab]
OR trial[tiab]
OR "controlled trial"[tiab]))
```

SPORTDiscus (n=69)

```
("non-alcoholic fatty liver disease"
OR "nonalcoholic fatty liver disease"
OR NAFLD
OR "metabolic dysfunction-associated steatotic liver disease"
OR MASLD
OR "non-alcoholic steatohepatitis"
OR NASH
OR MASH
OR "hepatic steatosis"
OR "liver fat")
AND
(exercise
OR "exercise training"
OR "physical training"
OR "aerobic training"
OR "resistance training"
OR "strength training"
OR "endurance training"
OR "high-intensity interval training"
OR HIIT
OR "combined training")
AND
(randomized OR randomised OR trial OR "controlled trial")
```

Web of Science (n=842)

```
(("non-alcoholic fatty liver disease"
OR "nonalcoholic fatty liver disease"
OR NAFLD
OR "metabolic dysfunction-associated steatotic liver disease"
OR MASLD
OR "non-alcoholic steatohepatitis"
OR "nonalcoholic steatohepatitis"
OR NASH
OR MASH
OR "hepatic steatosis"
OR "liver fat")
AND
(exercise
OR "exercise training"
OR "physical training"
OR "aerobic training"
OR "resistance training"
OR "strength training"
OR "endurance training"
OR "high-intensity interval training"
OR HIIT
OR "combined training")
AND
(randomized
OR randomised
OR randomly
OR random*
OR trial
OR "controlled trial"))
```

Cochrane CENTRAL (n=892)

```
("non-alcoholic fatty liver disease"
OR "nonalcoholic fatty liver disease"
OR NAFLD
OR "metabolic dysfunction-associated steatotic liver disease"
OR MASLD
OR "non-alcoholic steatohepatitis"
OR NASH
OR MASH
OR "hepatic steatosis"
OR "liver fat")
AND
(exercise
OR "exercise training"
OR "aerobic training"
OR "resistance training"
OR "strength training"
OR "high-intensity interval training"
OR HIIT
OR "combined training")
```

Scopus (n=1181)

```
TITLE-ABS-KEY (
"non-alcoholic fatty liver disease"
OR "nonalcoholic fatty liver disease"
OR NAFLD
OR "metabolic dysfunction-associated steatotic liver disease"
OR "metabolic dysfunction associated steatotic liver disease"
OR MASLD
OR "non-alcoholic steatohepatitis"
OR "nonalcoholic steatohepatitis"
OR NASH
OR "metabolic dysfunction-associated steatohepatitis"
OR "metabolic dysfunction associated steatohepatitis"
OR MASH
OR "hepatic steatosis"
OR "liver fat"
)
AND
TITLE-ABS-KEY (
exercise
OR "exercise training"
OR "physical training"
OR "aerobic training"
OR "resistance training"
OR "strength training"
OR "endurance training"
OR "high-intensity interval training"
OR HIIT
OR "combined training"
)
AND
TITLE-ABS-KEY (
randomized
OR randomised
OR randomly
OR random*
OR trial
OR "controlled trial"
OR "randomized controlled trial"
OR "randomised controlled trial"
)
```

Embase (n=1188)

```
('nonalcoholic fatty liver disease'/exp
OR 'non alcoholic fatty liver disease':ti,ab
OR 'nonalcoholic fatty liver disease':ti,ab
OR nafld:ti,ab
OR 'metabolic dysfunction associated steatotic liver disease':ti,ab
OR masld:ti,ab
OR 'nonalcoholic steatohepatitis':ti,ab
OR nash:ti,ab
OR mash:ti,ab
OR 'hepatic steatosis':ti,ab
OR 'liver fat':ti,ab)
AND
('exercise'/exp
OR 'exercise therapy'/exp
OR exercise:ti,ab
OR 'exercise training':ti,ab
OR 'aerobic training':ti,ab
OR 'resistance training':ti,ab
OR 'strength training':ti,ab
OR 'high intensity interval training':ti,ab
OR hiit:ti,ab
OR 'combined training':ti,ab)
AND
('randomized controlled trial'/exp
OR random*:ti,ab
OR trial:ti,ab
OR 'controlled trial':ti,ab)
```
